# Probiotics and Gut Microbiota in Obesity: Myths and Realities of a New Health Revolution

**DOI:** 10.3390/jpm12081282

**Published:** 2022-08-04

**Authors:** Xavier Eugenio León Aguilera, Alexander Manzano, Daniela Pirela, Valmore Bermúdez

**Affiliations:** 1 Departamento de Post-Grado, Universidad Católica de Cuenca, Ciudad Cuenca 010109, Ecuador; 2 Endocrine and Metabolic Diseases Research Center, School of Medicine, Universidad del Zulia, Maracaibo 4002, Venezuela; 3 Facultad de Ciencias de la Salud, Universidad Simón Bolívar, Barranquilla 080022, Colombia

**Keywords:** obesity, gut microbiota, short chain fatty acids, probiotics, prebiotics

## Abstract

Obesity and its comorbidities are humans’ most prevalent cardio-metabolic diseases worldwide. Recent evidence has shown that chronic low-grade inflammation is a common feature in all highly prevalent chronic degenerative diseases. In this sense, the gut microbiota is a complete ecosystem involved in different processes like vitamin synthesis, metabolism regulation, and both appetite and immune system control. Thus, dysbiosis has been recognised as one of the many factors associated with obesity due to a predominance of *Firmicutes*, a decrease in *Bifidobacterium* in the gut, and a consequent short-chain fatty acids (SCFA) synthesis reduction leading to a reduction in incretins action and intestinal permeability increase. In this context, bacteria, bacterial endotoxins, and toxic bacterial by-products are translocated to the bloodstream, leading to systemic inflammation. This review focuses on gut microbiota composition and its role in obesity, as well as probiotics and prebiotics benefits in obesity.

## 1. Introduction

Obesity is a chronic, complex, endocrine-metabolic disease that is a significant risk factor for numerous comorbidities [1]. Data from the National Health and Nutrition Examination Survey (NHANES) have shown that obesity prevalence in the United States increased from 30.5% in 1999–2000 to 42.4% in 2017–2018 [2], a fact that is conveyed in healthcare costs, not only in the USA but in many countries with Westernized lifestyles [3,4,5].

Some risk factors such as daily calories ingested, socioeconomic level, place of residence (urban or rural), sex, age, physical activity, diet quality, and behavioural disorders [6], among many others [7], drive obesity development throughout life in connection with genetic factors. Thus, scientific evidence has been accumulating regarding gut microbiota (dysbiosis) alterations can play a central role in the onset and further development of obesity, leading to coin the term “obese microbiota”, as that microscopic community of the distal digestive tract that swifts away from its typical architecture [8]. In this vein, the gut microbiota refers to the microorganism population that usually inhabits the intestinal lumen, which plays a pivotal role in the physiology, pathophysiology, and evolution of multiple diseases such as diabetes mellitus [9], irritable bowel syndrome [10], Parkinson’s disease [11], bronchial asthma [12], and even obesity [13], probably mediated by anti-inflammatory and immune response regulators metabolites [14,15]. Moreover, a significant proportion of individuals with obesity exhibit variations in their gut microbiota. There is scientific evidence enough to support that dysbiosis and inflammation play a pivotal role in many gastrointestinal and metabolic diseases, particularly obesity [16].

This review aims to briefly examine the intestinal microbiota structure, metabolism, and possible changes in obesity and the potential link with the endocrine-metabolic disorders usually associated with this condition. In addition, a critical analysis of first- and second-generation probiotics’ usefulness in obesity management is presented, as well as promising innovations in dysbiosis therapy and their possible impact on obesity prevention and treatment.

## 2. Structure of the Human Gut Microbiota

The microbiota refers to the set of bacteria, fungi, and viruses found in different tissues that, although individual, have a common characteristic in most microorganisms for a particular host. Eubiosis occurs when microbiota microorganisms are functional compositional and ecologically in equilibrium with the host [17]. The human microbiota comprises nearly 10,000 microorganism species and sub-species [18], with the gut microbiome containing over nine million genes [19]. *Firmicutes*, *Bacteroidetes*, *Actinobacteria*, *Proteobacteria*, *Fusobacteria*, and *Verrucomicrobia* are the main microorganisms in the gut microbiota, with a clear predominance of *Firmicutes* and *Bacteroidetes* representing 90% of the human gut microbiota [20].

**Figure 1:**
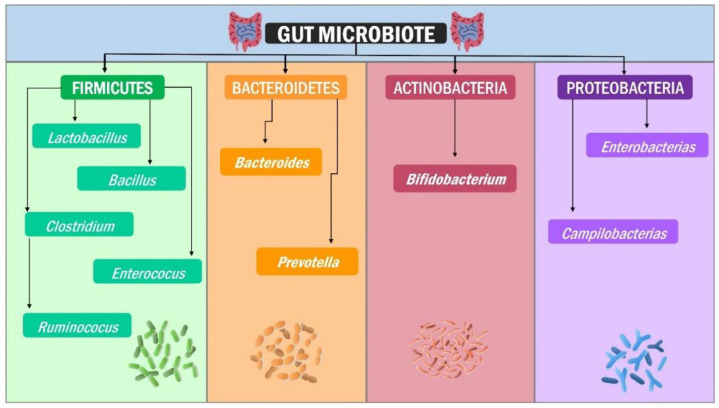
Gut microbiota composition: *Firmicutes*, *Bacteroidetes*, *Actinobacteria*, and *Proteobacteria* are the four “standard” *Phyla* in our gut intestinal microbiota. Each *phylum* comprises bacteria with diverse structures, metabolism, and functions.

The *phylum Firmicutes* comprises Gram-positive endospores-forming bacteria and a genome with low DNA G + C content [21]. More than 200 genera, including *Lactobacillus*, *Bacillus*, *Clostridium*, *Enterococcus*, and *Ruminicoccus*, have rigid or semi-rigid walls, with Clostridium accounting for 95% of them [22]. The *phylum Actinobacteria*, which consists of Gram-positive, branched, non-motile, non-spore-forming microorganisms, is the least predominant at the intestinal level and is represented by the *genus Bifidobacterium* [23] and plays a vital role in the colonisation of the digestive tract of children born by vaginal delivery, conversely, it is significantly less in those born by cesarean section [24]. On the other hand, the *phylum Bacteroidetes* is composed of Gram-negative bacteria of *Bacteroides* and *Prevotella* genera [20], playing as primary polysaccharide processors [25] and whose final products are substrates for enzymes from other bacterial genera that eventually participate in the immune system regulation [26], intermediary metabolism [27] and intestinal–brain axis signalling [28,29,30]. Bacteria such as *Lactobacillus (Firmicutes)* and *Bifidobacterium (Actinobacteria)* hydrolyse and ferment very specific polysaccharides to produce the short-chain fatty acids (SCFA) acetate, butyrate, and propionate (Figure 1) [31]. In this regard, propionate is produced mainly by *Akkermansia muciniphila*, whereas butyrate is produced by *Faecalibacterium prausnitzii*, *Eubacterium hallii*, and *Eubacterium rectale* [32].

## 3. Are the SCFA the Missing Link between Dysbiosis and Obesity?

SCFAs are G protein-coupled receptors (GPCRs) ligands, activating anti-inflammatory signaling cascades [33]. In addition, SCFA may also act by preserving intestinal integrity [34], reducing luminal pH [35,36], protecting intestinal lining, especially the luminal mucopolysaccharide layer [37], and regulating GAP and tight junction expression.

Although diffusion of short-chain fatty acids in their neutral (undissociated) form is an important absorption mechanism, anionic form uptake by transporter proteins is the main route for SCFA translocation across the cell membrane. Several transport systems have been described for SCFA transport in colonocytes: MCT1 (SLC16A1) and MCT4 (SLC16A3) are H+ coupled electroneutral transporter. MCT1 is expressed in the apical and basolateral membrane of the colonic epithelium, whereas MCT4 is specifically expressed in the basolateral membrane. SMCT1 (SLC5A8) is a Na+ a coupled electrogenic transporter [38], whilst SMCT2 (SLC5A12) is a Na+ a coupled transporter but electroneutral. SMCT1 and SMCT2 are expressed exclusively at the apical membrane [39,40]. Finally, an anion exchange mechanism coupled to bicarbonate efflux has been reported in the literature, but their identification and isolation have remained elusive. As a result of these mechanisms, SCFAs are efficiently absorbed across the colonocyte apical membrane in approximately 90–95% [41].

The downstream SCFA effects are mediated, at least in part, by histone deacetylase (HDAC) inhibition [42] downstream and thus decrease pro-inflammatory cytokine production. In addition, other essential functions of SCFA involve the peroxisome proliferator-activated receptor-alpha activation, increasing the substitution of old cells by renewed epithelial colonic cells [43] and the induction of IL-10 in T cells, as well as increasing the antibodies production through B cells differentiation into plasma cells [44] improving the adaptative immunity response [45]. In addition, factors such as SCFA speed, volume, and acetate/propionic/butyric ratio bring a complex interplay between fermentable diet polysaccharides, microbiome diversity and activity, and, eventually, the gut transit time.

**Figure 2:**
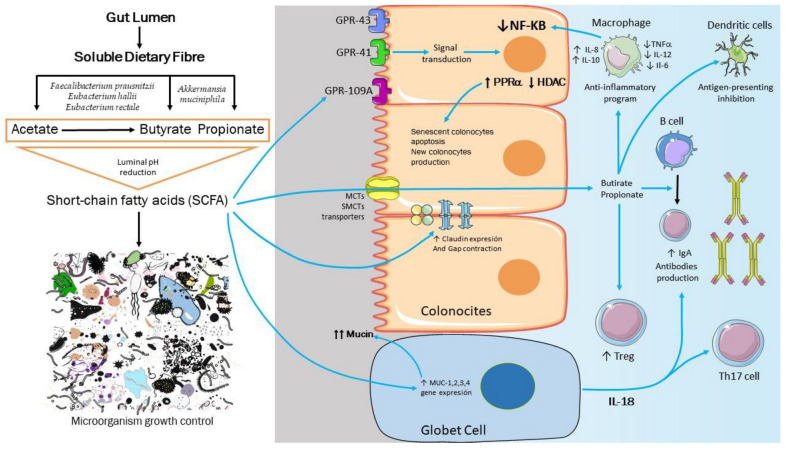
Main effects of short-chain fatty acids (SCFA) on microbiota and epithelium barrier. SCFA protect the intestinal barrier by lowering the levels of TNFα and interleukin (IL)-6, activating the G-protein coupled receptors (GPCR) to participate in PParα expression increase and the intestinal-mediated inflammatory and immune response by suppressing histone deacetylase (HDAC) and downregulating the expression of pro-inflammatory cytokines. Additionally, SCFA upregulates the gene expression of mucin family genes (MUC1–4) in the intestine, and the protons generated by SCFA dissociation produce an osmotic pressure imbalance in the bacteria. Furthermore, SCFA inhibits bacterial multiplication by interfering with DNA and protein synthesis.

It is well-known that fermentable carbohydrate consumption and SCFA administration produce a wide range of health benefits, including body composition improvement, increased insulin sensitivity, a low-risk lipid profile, body weight reduction, and cancer risk reduction as colon cancer. In the same vein, SCFA also plays an essential role in obesity pathophysiology by intermediary metabolism regulation and appetite control [46], at least, in part, by stimulating the incretins synthesis and secretion by entero-endocrine L cells, a specific butyrate-mediated effect [47,48]. Thus, an interesting group of scientific papers conducted in humans by Chambers et al. [49] using soluble fiber as inulin propionate-ester demonstrated an appetite and food intake attenuating effect mediated by propionate. Other studies from the same group have found equivalent results regarding food choice [50], improving pancreatic function and modulation of liver lipid metabolism. Moreover, oral propionate administration increases fat oxidation in humans regarding energetic metabolism regulation. Finally, in experimental models, propionate reduces food intake via the gut–brain axis in animals [51]. This evidence highlights an SCFA-rich diet’s importance in managing obesity and associated diseases, including hypertension and other cardiovascular diseases. For instance, the butyrate induces renal prorenin receptors and renin expression, attenuating the angiotensin-II effects [52], and acetate and propionate regulate renin production via Olfr78 and a counter-regulation loop with FFAR3 [53] (Figure 2).

## 4. Molecular Basis of Intestinal, Immunological, and Metabolic Homeostasis Control by Gut Microbiota

Dietary fiber is a sundry group of complex carbohydrates affecting human metabolism and gut microbiota [54]. Arabinoxylans (AX) are cell wall components that constitute a significant part of the dietary fiber fraction found in cereals and represent a significant fiber source in the human diet [55]. These heteroxylans, especially AX, deserve special attention with cellulose, β-1,3;1,4-glucan, arabinogalactan peptide, and lignin, as they are the major components of fiber in cereals [56].

**Figure 3:**
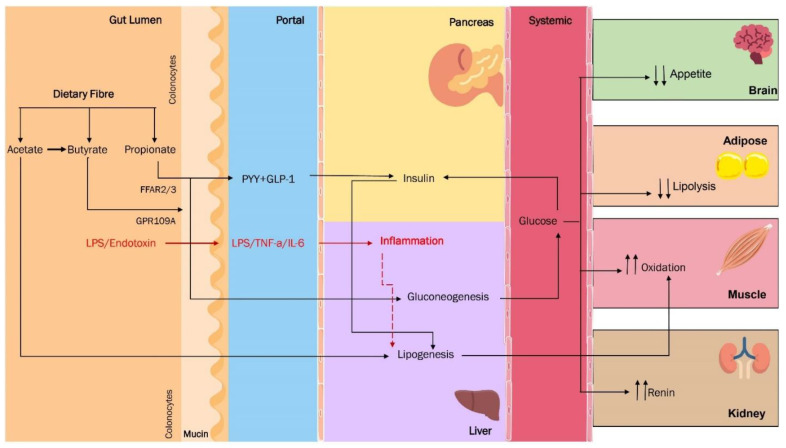
Dietary fibre and its effects on human metabolism and gut microbiota.

Bifidobacteria metabolise non-digestible carbohydrates to produce acetate and lactate. Some studies have screened for genes encoding several putative AXOS-degrading enzymes in a wide Bifidobacteria variety species, but no clear correlation has been made regarding these polysaccharides’ degradation [57,58]. The primary site for inulin fermentation is the ascending colon. In contrast, AXOS fermentation occurs in the transverse colon [59], and *Bifidobacterium*, *Bacteroides*, and *Roseburia* have been identified as the main fermenting species of these carbohydrates (Figure 3) [60].

A high-fat diet has been established to increase Clostridia and Bifidobacteria gut population and decrease *Bacteroides*. However, there is also a significant influence on bile acid metabolism [61], highlighting its potential impact on dysbiosis, which might lead to changes in SCFA metabolism due to *Bifidobacteria* decrease.

## 5. Microbiota in Obesity: Cause, Effect, or Both?

Dysbiosis refers to the alteration of normal bacterial microbiota [62]. Several environmental factors affecting gut microbiota are closely associated with dysbiosis and obesity [63]. Overall, these factors increase *Firmicutes* species such as *Eubacterium rectale*, *Clostridium coccoides*, *Lactobacillus reuteri*, *Clostridium histolyticum*, and *Staphylococcus aureus* [64]. On the other hand, a significant decrease in the relative abundance of several Bacteroidetes taxa members such as *Faecalibacterium prausnitzii*, *Bacteroidetes*, *Methanobrevibacter smithii*, *Lactobacillus plantarum* and *Lactobacillus paracasei*, *L. rhamnosus*, and *Verrucomicrobia (Akkermansia muciniphila)* have been reported [16,65]. Depending on the microbiota alteration, dysbiosis can be produced by the loss of beneficial microbial organisms, overgrowth of potentially harmful microorganisms, or general microbial diversity loss [66]. Thus, obesity microbiota is characterised by a significant *Firmicutes* increase and a 50% decrease in Bacteroidetes at the terminal ileum [67]. It was also reported that *Lactobacillus reuiteri* and *Lactobacillus sakei* are directly related to weight gain in adults, mainly in conjunction with a high-fat diet consumption [68], while on the other hand, microorganisms of the *Bifidobacterium* genus decrease the comorbidities associated with obesity, such as metabolic syndrome [69].

High-fat and high-carbohydrate diet consumption leads to *Bacteroides*, *Bifidobacterium*, *Lactobacillus*, and *Akkermansia* deficit with a considerable increase in *Clostridium* and *Prevotella* driving Toll-like receptor 4 (TLR4) activation via lipopolysaccharides pathway triggering to inflammation and claudin 1 and 3 expression decrease [70]. Claudin is a family of 18 proteins that play essential structural and functional roles in determining tight junction structure and permeability. Therefore, a reduction in these protein’s expression via SCFA levels decrease alters the integrity gut epithelial barrier, causing bacterial translocation and expression of inflammatory cytokines [71] such as TNFα and interleukin-1 [72], increasing Gram-negative bacteria adherence to the intestinal epithelium [73], leading to a chronic pro-inflammatory state that triggers obesity and cardiometabolic comorbidities [74]. Furthermore, it has been recognised that high-glucose or fructose rich-foods produce microbiota dysbiosis, also affecting intestinal permeability and inducing hepatic steatosis [75], while on the other hand, glutamine, vitamin A, vitamin D, and dietary fibre preserve intestinal permeability by increasing the production of butyrate and propionate [76].

## 6. Are Probiotics Effective in Endocrine-Metabolic Disease Treatment? Are These Supplements Effective in Intestinal Dysbiosis?

According to The World Health Organization, probiotics are *“Live microorganisms which when administered in adequate amounts confer a health benefit on the host”* [77]. In this area of intense development, scientific evidence has been accumulated, influencing the appearance of new, rich, and sophisticated terminology. Since it has been shown that inactivated, non-viable, or broken probiotics have beneficial effects on health, the term paraprobiotics has been incorporated, including the true probiotics (functional), pseudoprobiotics (inactive), and phantom probiotics (non-viable or broken) [78]. Thus, a true probiotic, for instance, *Lactobacillus plantarum* improves IL-10 in the colon [79] by adhering to the gut epithelium. *Lactobacillus rhamnousus*, adhering to the epithelium and, in this case, also producing lactic acid [80]; Lactobacillus acidophilus is found at the intestinal level and whose mechanism of action is by lactacin production and further cholesterol adherence [81]. *Bifidobacterium animalis* increase intestinal motility and bile salts hydrolysis [82]. Furthermore, *Saccharomyces boullardii* redistributes T cells in vitro (Table 1) [83].

There is a growing body of evidence that probiotics improve, maintain, or restore the intestinal microbiota, a critical action because obesity has been linked to dysbiosis [84], thus opening the door to innovative manoeuvres directed to microbiota architecture and diversity.

## 7. Efficacy of Probiotics in Obesity and Its Comorbidities: Fiction or Reality?

The benefit of probiotics in overweight and obese patients has long been controversial. However, most research suggests limited health benefits, so they are only recommended as adjuvant therapy for cardiovascular disease biomarkers reduction [85].

In this regard, a meta-analysis by Koutnikova et al. on 105 trials assessing the probiotic’s efficacy in obese patients concluded that while probiotics improved BMI and weight by 3–5%, there was no statistically significant effect on HbA1c, cholesterol, triglycerides, HOMA-IR, or liver function [86]. Conversely, a recent meta-analysis of randomised controlled trials (RCTs) by Tabrizi et al., assessing probiotic effectiveness on clinical symptoms, weight loss, glycemic control, blood lipids, endocrine profiles, inflammation biomarkers, and oxidative stress tests in women with polycystic ovary syndrome, found improvements in women consuming probiotics related to weight, BMI, FPG, insulin, HOMA-IR, triglycerides, VLDL-cholesterol, C reactive proteín, malondialdehyde, hirsutism, total testosterone, QUICKI, nitric oxide, total antioxidant capacity, reduced glutathione, and sexual hormones binding globulin. However, it did not find changes in dehydroepiandrosterone sulfate levels and total cholesterol, LDL, and HDL cholesterol levels in patients with PCOS [87].

Similarly, a meta-analysis by Lau et al. evaluated the probiotic effect on obesity, type 2 diabetes, hypertension, and dyslipidemia in 38,802 adults from the National Health and Nutrition Examination Survey (NHANES) data between 1999 and 2014. In this research, Probiotic ingestion was considered when a subject reported yoghurt consumption or when a commercial probiotic supplemented it during the 24-h dietary recall or the Dietary Supplement use 30-Day questionnaire. This study found a 13.1% reported probiotic ingestion in the participants. The prevalence of obesity and hypertension was lower in the probiotic group (obesity-adjusted Odds Ratio (OR): 0.84, 95% CI 0.76–0.92, *p* < 0.001; hypertension-adjusted OR: 0.79, 95% CI 0.71–0.88, *p* < 0.001). Accordingly, even after analytic adjustments, body mass index (BMI) was significantly lower in the probiotic group, as were systolic and diastolic blood pressure and triglycerides; high-density lipoprotein (HDL) was significantly higher in the probiotic group for the adjusted model [88].

In an interesting meta-analysis conducted by Kim et al. with data from 1953 and 2018, including 246 obese cases and 198 normal controls, the primary goal was to determine whether SCFA levels differ between obese and non-obese individuals and determine their faecal microbiota structure. This study found differences in the levels of SCFAs in faeces between obese cases and non-obese controls. The findings show that individuals with obesity had higher acetate, propionate, and butyrate faecal levels. In addition, this study found that obese individuals had low bacterial abundance in faeces regarding microbiota architecture, but the differences were not statistically significant. The meta-regression analysis demonstrated that the abundance of the phylum *Firmicutes* was positively associated with obesity for individuals 37 years or younger, while the Bacteroidetes abundance was negatively associated with obesity for 47 or younger participants.

## 8. Prebiotics, the Cornerstone of Gut Microbiota

Since the prebiotic concept has experienced a continuous evolution over decades, the classic definition proposed by Gibson et al. is *“a non-digestible food ingredient that beneficially affects the host by selectively stimulating the growth and/or activity of one or a limited number of bacteria in the colon*, *thereby improving host’s health”* [89] still being useful to understand the fundamental properties of this substances. This concept has become more sophisticated to include noncarbohydrate substances affecting the colonisation of beneficial bacteria within our microbiota. Prebiotics are non-digestible oligosaccharides with diverse chemical structures and functions, differing in molecular weight, monosaccharide type, origin (vegetables, fruits, among others), and branching degree. Prebiotics are currently a new and flourishing research area with a profound impact as modulators of gut microbiota and their effects on cancer, ulcerative colitis, irritable bowel syndrome, type 2 diabetes, response to chemotherapy, immune modulation, and obesity, among others. Prebiotics exert beneficial effects based on stimulating the growth of colon probiotics and beneficial taxons with many anti-inflammatory capacities and in their catabolic products, especially butyrate, which can carry out a diverse and complex cell cycle regulation in colonic cell tumour, leading towards their apoptosis [90].

Prebiotics has demonstrated their usefulness in regulating satiety by increasing GLP-1 and PYY [91], as well as decreasing ghrelin secretion [92], which results in a reduction in food intake and consequent weight loss [93]. Nutritionally, AX are the main component of dietary fiber, and their enzymatic hydrolysis produces AXOS and XOS; which, when cereal-based foods, such as bread and beer, are consumed, exhibit all of the characteristics of prebiotics, including resistance to gastric acidity, fermentation by the intestinal microbiota, and selective stimulation of the growth or activity of beneficial bacteria [55]. As a result, AXOS and XOS selectively stimulate *Bifidobacterium*, which stops the increase in cholesterol, triglycerides, postprandial glucose, and insulin levels [94].

## 9. Concluding Remarks

Humans have not escaped from the co-evolution with the vast microbial community residing in our bodies. As a superorganism, the mammalian gut is an excellent niche for microbes because of its constant temperature, predictable humidity, pH, and steady food supply. In return, the microbes perform a pleiad of functions ranging from vitamin production to immune system regulation and appetite control.

Until now, we had never had enough knowledge to modify this interaction to our benefit since 10,000 years ago, when the most significant change in the host-microbiota symbiosis occurred during the Neolithic revolution, a transition from hunting and gathering to agriculture and permanent settlement. In this period, agriculture and animal husbandry began to shape the genomes within us in an accelerated and non-stop fashion. The second giant leap in this relationship occurred during the industrialisation process and, most recently, during the Cocacolonization [95] and McDonalization [96] era, at least in the last five decades, driving dramatic changes in the human gut microbiota structure as we see in people with obesity, diabetes and other chronic diseases in the 20th and 21st century.

While we cannot entirely deny that the rationale for probiotics administration seems solid, it is also clear that we have a long way to go in understanding both the microbiota complexity and probiotics’ effects on many diseases, including obesity. Each of us has a unique gut microbiome, and thus, the effects of the different bacteria in commercial probiotics can be highly variable; therefore, we believe their use should be tailored within a framework of personalised medicine that takes into account the disease to be treated and the microbiota affected by that particular patient for optimal benefits. Thus, it is almost certain that current commercial products contain neither the correct strains nor the correct amounts of bacteria to provide benefits for most of the diseases to be treated. Therefore, while taking a supplement to improve health is undoubtedly an attractive prospect, there are no robust controlled clinical trials aimed at assessing the efficacy of probiotics as individual agents for treating obesity, as we have seen in dozens of anti-obesity drugs that have been developed over the decades. Until now, those patients seeking to improve chronic diseases such as obesity by helping their gut microbiota with probiotics pursue a mirage under a promise from the manufacturing companies that take advantage of not having to substantiate their claims with randomised controlled trials. Faced with this knowledge gap, it should consider consuming a healthy diet rich in soluble fibre from fruits and vegetables. In the meantime, rigorous clinical trials with very high-resolution genomic analysis are needed to corroborate the potential health benefits and confirm whether all the effects attributed to probiotics from the supplement industry are simply a myth or a new health revolution helping us stop the ominous obesity epidemic.

## Figures and Tables

**Table 1:** The major species used as probiotics for the gastrointestinal tract in humans and their main locations and mechanisms of action [78].

Microorganism	Location	Mechanism of Action
*Lactobacillus plantarum*	Gastrointestinal tract	Adhesion to epithelial cells Improves IL-10 production in the colon
*Lactobacillus rhamnosus*	Gastrointestinal tract and brain	Adhesion to epithelial cells, lactic acid production, Regeneration of epithelial cells, increases GABA receptors in cortical regions and decreases in the amygdala, hypothalamus, and *Locus ceruleus*
*Lactobacillus salivarius*	Gastrointestinal tract	Secretion of low molecular weight bacteriocins.
*Lactobacillus reuteri*	Gastrointestinal tract	Reuterin (3-hydroxypropionaldehyde) production
*Lactobacillus acidophilus*	Intestine	Cholesterol adherence
*Lactobacillus casei*	Intestine	Inhibits bacterial translocation, increases MUC gene expression, inhibits cholesterol mycelia formation, enhances NK cell activity, inhibits bacterial translocation, increases MUC gene expression, inhibits cholesterol mycelia formation, and enhances NK cell activity
*Lactobacillus bulgaricus*	Intestine	Adherence to cholesterol, inhibits the formation of cholesterol mycelia.
*Bifidobacterium longum*	Intestine	Binding to aflatoxin B1
*Bifidobacterium adolescentis*	Intestine	Binding to aflatoxin B1
*Bifidobacterium animalis*	Intestine	Increases intestinal motility and bile salts hydrolysis.
*Saccharomyces boullardii*	Intestine	T-cell redistribution
*Lactobacillus plantarum*	Intestine	Decreased translocation and adherence of pathogens
*Lactobacillus paracasei*	Intestine	Enhances cancer cell apoptosis
*Saccharomyces boulardii*	Intestine	Improves intestinal barrier function
*Bifidobacterium* sp.	Intestine	Secretes superoxide dismutase
*Lactobacillus helveticus*	Intestine	Secretes vitamin E

## Data Availability

Not applicable.
